# Development and application of a rapid rehabilitation system for reconstruction of maxillofacial soft-tissue defects related to war and traumatic injuries

**DOI:** 10.1186/2054-9369-1-11

**Published:** 2014-06-01

**Authors:** Shi-zhu Bai, Zhi-hong Feng, Rui Gao, Yan Dong, Yun-peng Bi, Guo-feng Wu, Xi Chen

**Affiliations:** State Key Laboratory of Military Stomatology, Department of Prosthodontics, School of Stomatology, the Fourth Military Medical University, 145 Changlexi Road, Xi’an, Shaanxi, 710032 China

**Keywords:** CAD/CAM, Maxillofacial defect, Prosthesis design, Defect rehabilitation, War and traumatic injuries

## Abstract

**Background:**

The application of a maxillofacial prosthesis is an alternative to surgery in functional–aesthetic facial reconstruction. Computer aided design/computer aided manufacturing has opened up a new approach to the fabrication of maxillofacial prostheses. An intelligentized rapid simulative design and manufacturing system for prostheses was developed to facilitate the prosthesis fabrication procedure.

**Methods:**

The rapid simulation design and rapid fabrication system for maxillofacial prostheses consists of three components: digital impression, intelligentized prosthesis design, and rapid manufacturing. The patients’ maxillofacial digital impressions were taken with a structured-light 3D scanner; then, the 3D model of the prostheses and their negative molds could be designed with specific software; lastly, with resin molds fabricated by the rapid prototyping machine, the prostheses could be produced directly and quickly.

**Results:**

Fifteen patients with maxillofacial defects received prosthesis rehabilitation provided by the established system. The total clinical time used for each patient was only 4 hours over 2 appointments on average. The contours of the prostheses coordinated properly with the appearance of the patients, and the uniform-thickness border sealed well to adjacent tissues. All of the patients were satisfied with their prostheses.

**Conclusions:**

The rapid simulative rehabilitation system of maxillofacial defects is approaching completion. It could provide an advanced technological solution for the Army in cases of maxillofacial defect rehabilitation.

## Background

War and traumatic injuries may result in maxillofacial soft-tissue defects and deformities; for instance, auricular, nasal, or orbital defects or complex multi-organ defects and deformities [1]. Defects in the maxillofacial region have severe and complicated impacts on the patient’s physical and mental health, leading to deficits in functions such as mastication, speech, and deglutition. Moreover, such injuries also result in serious facial deformations, thus handicapping the patient’s daily activities. Some patients who have suffered maxillofacial defects may show suicidal tendencies, and the army’s morale and ability to recruit new soldiers may be adversely affected. Thus, it is of great significance in the field of military medicine to rehabilitate and reconstruct the lost function and damaged appearance caused by maxillofacial war injuries and help these patients return to society.

Surgery was routinely performed to reconstruct maxillofacial defects and deformities. However, the normal appearance and structure of the facial region is often beyond the capability of surgical reconstruction, considering the subtle and intricate characteristics of the local tissue and structures. The application of a maxillofacial prosthesis is an alternative to reconstructive surgery, either because of the poor psychophysical condition of the patient or because of excessive tissue loss [2]. However, the traditional method of fabricating maxillofacial prostheses includes several complex steps; it is a labor-intensive and time-consuming task, and the final results mainly depend on the experiences and skills of the clinician [3].

The application of computer aided design/computer aided manufacturing (CAD/CAM) in this field has opened up a new approach to the fabrication of maxillofacial prostheses [4–10]. The School of Stomatology, Fourth Military Medical University has been researching this field since 2001 and has developed an intelligentized rapid design and manufacturing system for maxillofacial prosthesis fabrication. The system consists of three components: digital impressions, simulation design of the prosthesis, and rapid manufacturing of the prosthesis.

## Methods

This study was carried out at the Department of Prosthodontics of the School of Stomatology, the Fourth Military Medical University, Xi’an. This hospital provides treatment for both military and civilian patients. The study was approved by the Institutional Review Board of the School and was conducted in accordance with the principles of the Declaration of Helsinki. A clinic assistant informed all subjects about the nature of the study. Informed consent was obtained from each subject who agreed to participate in the study.

### Maxillofacial digital impression

Digital data were obtained by a structured-light three-dimensional (3D) scanning system and a computed tomography (CT) scanning system.

#### Structured-light 3D scanning

**Figure 1:**
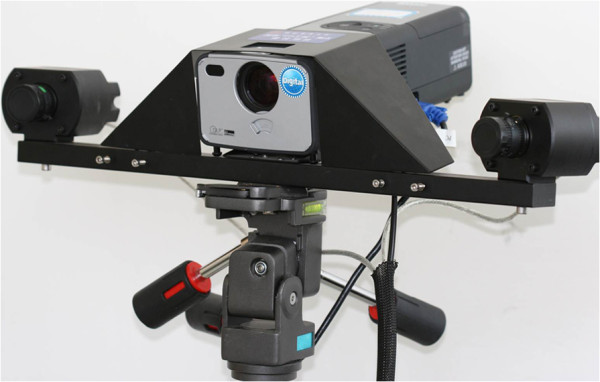
**Structured-light 3D scanner.** The device consists of two charge-coupled device cameras and one projector.

**Figure 2:**
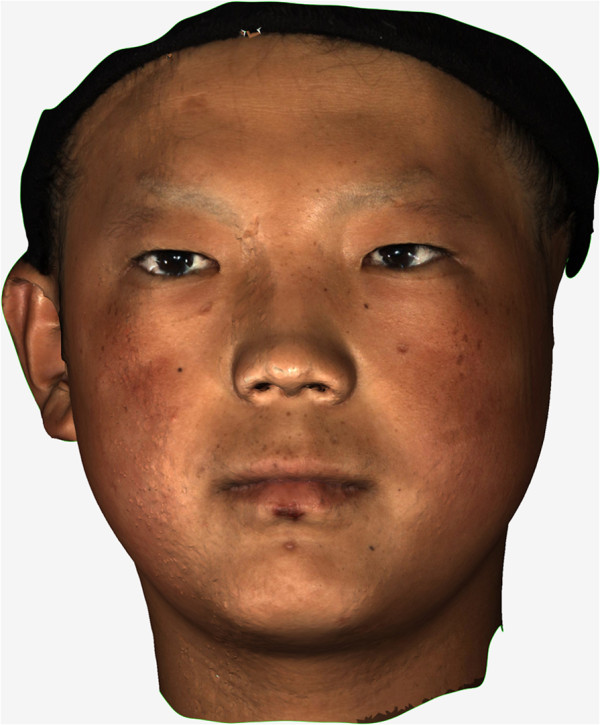
**3D model of the patient.** Original 3-dimensional (3D) facial image of the patient was generated from data from the 3D scanner.

The 3D sensing scanning technique integrated several 3D non-contact measurement techniques, such as the structured light technique, the phase measurement technique, and the computer vision technique. Several narrow stripes of light were projected onto the surface of the target object and produced lines of illumination that appeared distorted from other perspectives than that of the projector, so an exact geometric reconstruction of the surface shape could be reconstructed. The 3D data for the patient’s facial surface were obtained in a point-cloud format by a structured-light 3D scanner (3DSS-STD-II, Digital Manu, Shanghai, China) consisting of two charge-coupled device cameras and one projector connected to a personal computer (Figure 1).Before scanning, the patient was asked to sit up straight and keep a stabilized facial expression with the mouth closed naturally. The camera position and shutter were adjusted to the patient’s face, and scanning was carried out from in front of and 30 degrees to the right and left of the patient. Data were saved in the point-cloud format and imported to software that permitted optical representation of the surface and reconstruction of a 3D digital model (Figure 2).

#### Computed tomography scanning

**Figure 3:**
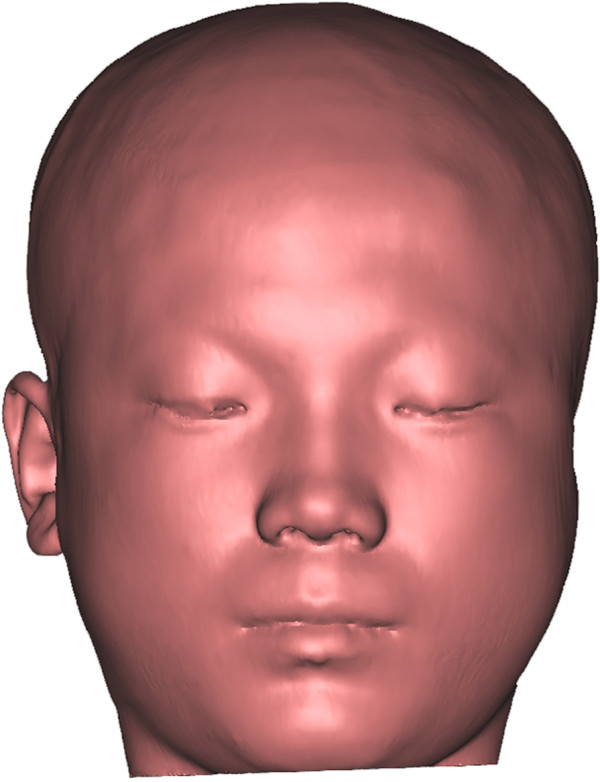
**CT scan model of the patient.** Maxillofacial digital model of the patient reconstructed from CT scan data.

An advantage of computed tomography (CT) scanning is the ability to simultaneously obtain the accurate morphology of the soft tissue and the hard tissue, thus remedying the flaw of invisible areas, such as ears, in structured-light 3D scanning. In China, optical scanning devices have not yet gained popularity in medical institutions, so CT scanning is still the dominant method to obtain 3D data for CAD/CAM-assisted prosthesis fabrication and is especially indispensable in auricular prosthesis fabrication.The patient was asked to lie down and keep a natural facial expression with his/her eyes open. The CT (Brilliance; Philips Healthcare, Andover, MA) scanning conditions should be: voltage 120 kV, current 150 mAs, thread pitch 1 Q, and slice thickness 2.5 or 1.25 mm. Continuous helical scanning started from the level of the inferior margin of the chin and stopped at the level of the calvarium with a slice thickness of 1.25 mm. Scanning data were saved in the DICOM format and imported to software to reconstruct the digital maxillofacial model (Figure 3).

### Intelligentized simulation design of maxillofacial prosthesis

Intelligentized simulation design of maxillofacial prosthesis is the most crucial step in the whole process and has a direct correlation with the definitive prosthetic treatment outcome. Nasal, auricular, and orbital prostheses are the most commonly observed prosthesis types in the clinic, and the design methods differ from each other due to their diverse features. Our system has developed a design process for every type of prosthesis, and auricular prostheses will be used as an example in this study.

#### Mirroring of the healthy ear

The 3D head model of the patient was oriented to the natural head position and served as a reference throughout the planning process [11]. The volume of the auricular prosthesis and its optimal position were then simulated in the software. The mid-plane was generated through the nasion, pronasale, and gnathion. The 3D image of the healthy ear was separated and mirrored at the defect site to create a pattern for the lost ear.

#### Adjustment of the position of the mirrored ear

The optimal position of the pattern was determined in relation to the patient’s face to the satisfaction of the patient and his/her relatives. The anteroposterior position, protrusion, inclination, and level of the pattern were compared with those of the normal ear; the prosthesis was viewed from the front, side, rear, and top of the defect to assess symmetry with the contra-lateral ear; and the tragus and the external meatus were also used as references at this point.

#### Design of the prosthesis pattern

**Figure 4:**
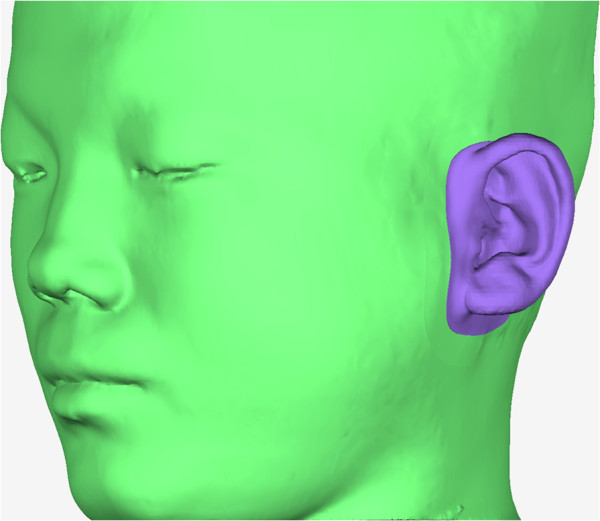
**Auricular prosthesis pattern.** The definitive prosthesis pattern was created in the system software with smooth margins, the width and thickness of which were precisely designed.

The covering range of the prosthesis was defined and separated from the soft tissue model by drawing a closed spline on the defect area. Another spline was then generated inside at a 0.5 mm distance from the first spline. The triangles between these two splines were selected out and offset 0.2 mm upwards, and then the outer boundary of the offset triangles was connected with the boundary of the covering range. The inner boundary of the offset triangles was projected onto the primitive pattern to generate a closed boundary line. On the pattern, the triangles encircled by this boundary line were selected out; then, this boundary was connected with the inner boundary of the offset triangles; next, the step-like line connecting these triangular patches was smoothed. The definitive pattern was created with a smooth margin that was exactly 0.2 mm in thickness and 0.5 mm in width (Figure 4).

#### Design of the negative mold

**Figure 5:**
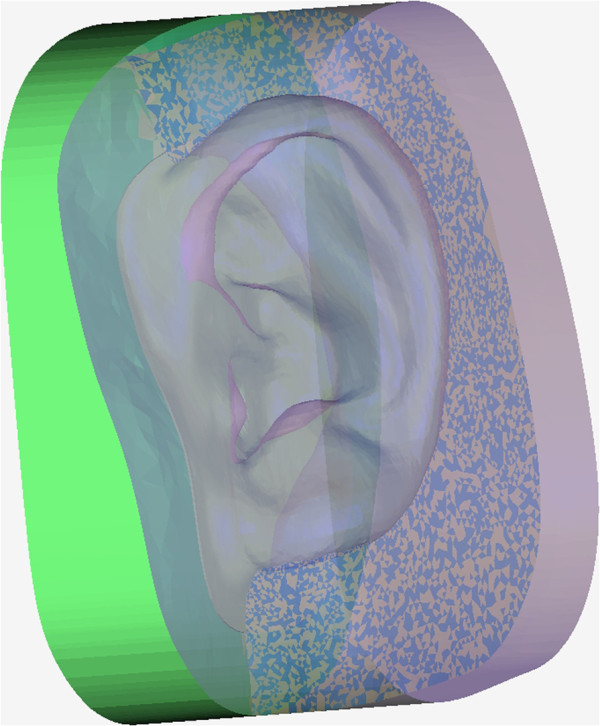
**Three-piece negative mold.** Lower and upper pieces of the negative mold were designed; the internal space of the auricular prosthesis is shown after assembling these pieces.

The ear pattern was transferred to a negative volume in the software. The outer boundary of the adhesive region was expanded outwards 10 mm on the defect area. The inner boundary of the adhesive region was connected to the internal surface of the prosthesis pattern, and the border of the expanded area was extruded 10 mm forward along the transverse axis. After closing the extruded bottom, the lower piece of the negative mold was obtained. The polygons of the expanded area were duplicated and bridged with the boundary of the external surface, and then the outer border of those polygons was extruded 40 mm backward along the transverse axis; the upper piece of the mold was obtained after closing the boundary. The prosthesis mold was separated into three parts, just as with conventional three-piece molds prepared in flasks, and the reference mortise and tenon joints were added to guarantee a secure seal of the three-part mold during the silicone processing procedures. These pieces were saved in STL format (Figure 5).

### Rapid fabrication of maxillofacial prosthesis

**Figure 6:**
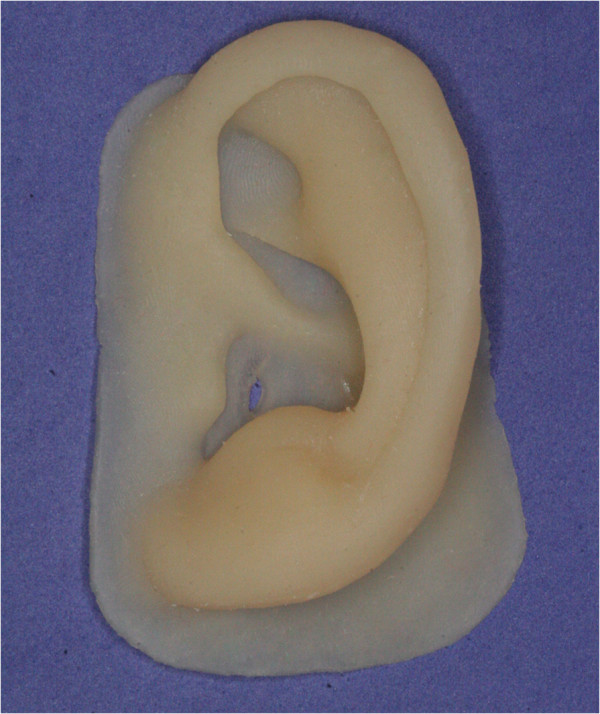
**Silicone elastomer prosthesis before extrinsic coloring.** Resin pieces of the mold were fabricated with the RP machine. A silicone prosthesis was obtained using routine procedures.

**Figure 7:**
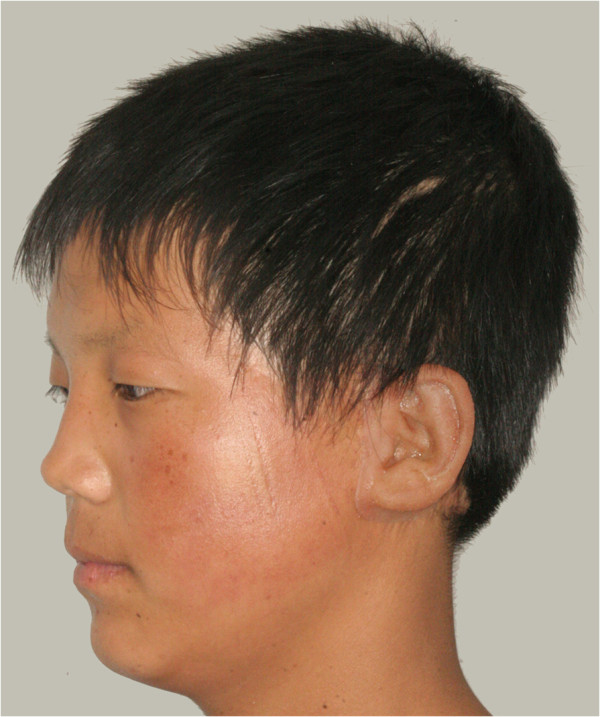
**Final result of rehabilitation.** A definitive silicone prosthesis was generated after applying extrinsic coloring and was applied to the patient.

After creating the virtual design of the maxillofacial prosthesis and its negative mold, the definitive prosthesis was fabricated. Our system used the method of direct fabrication of silicone elastomer prostheses by resin negative molds. The resin negative mold was made, and then silicone elastomer was filled into the negative mold to prepare the definitive prosthesis.Resin pieces of the mold were fabricated with a selective laser sintering (SLS) rapid prototyping machine (AFS-360; Longyuan Automated Fabrication System, Beijing, China). As with the flask in the conventional method, the negative mold was used to fabricate the definitive silicone elastomer auricular prosthesis (A-RTV-40; Factor II Inc, Lakeside, AZ) (Figure 6). Finally, extrinsic coloring was applied, and the definitive silicone prosthesis was applied to the patient (Figure 7). Sufficient retention was obtained using a prosthetic adhesive (Daro Adhesive Extra Strength, Factor II, Ariz, US).

### Establishment of the 3D nasal morphological database

In the design of prosthetics for organs with bilateral symmetry, the mirrored data from healthy side can easily be used; however, it is difficult to find a proper data source for nasal defects. A 3D model database of normal nasal shapes was established in the system to meet the needs of nasal prosthesis design.

#### Acquisition of 3D nasal morphological data

One thousand nasal models were collected by 3D reconstruction of existing CT scan data, and 200 nasal models were collected by structured-light 3D scanning. All of the models were saved as .STL files.

#### Development of database software

The database software was based on Microsoft Office Access and was written in Microsoft Visual C++. OpenGL was used to realize the visualization of the 3D data. Multiple nasal shape classifications were integrated to develop the database so that nasal models could be imported into the database accompanied by their morphological features, and the proper models for certain needs could also be easily found.

#### Application of the database

The feature classifications of the patient’s nose were defined according to photographs taken before the injury. After the corresponding classifications were entered into the search field of the database, the proper nasal model could be selected automatically for nasal prosthesis design. Use of the database was an efficient solution for the lack of data sources and was an indispensable step in nasal prosthesis design.

## Results

Between 2009 and 2013, maxillofacial prostheses were made with the intelligentized rapid design and manufacturing system for 15 patients, including 9 men (60%) and 6 women (40%). Their ages ranged from 23 to 59 years, with a mean of 49.3 ± 11.6 years. Facial defects were primarily the result of tumor resection (n = 6, 40%); additionally, 33% were the result of congenital defects (n = 5), and 27% were the result of acquired trauma (n = 4). The locations of the facial prostheses were distributed among auricular prostheses (n = 6, 40%), orbital prostheses (n = 5, 33%), and nasal prostheses (n = 4, 27%). All patients chose to accept an adhesive-retained prosthesis.

The total clinical time used for each patient was, on average, only 4 hours over 2 appointments. The patients’ maxillofacial digital impressions were taken at the first appointment, and then the primitive prosthesis pattern could be created and modified. The patient could confirm his or her acceptance of the aesthetic effect at the very beginning of the process. The next appointment was used for extrinsic coloring and distribution. In addition to this time, approximately 10 hours on average was needed to complete the design and fabrication processes. With the specific software, the design of the definitive prostheses and their negative molds could be accomplished in approximately 3 hours. The remaining time was used for rapid prototyping fabrication of the resin mold and for silicone processing.

The contours of the prostheses coordinated properly with the appearance of the patients, and the borders were sealed well to the adjacent tissue with uniform thickness. All of the patients were satisfied with the appearance of the prostheses designed and fabricated with this system.

## Discussion

The traditional method of fabricating prostheses involves taking facial impressions and measurements, sculpting wax patterns, try-in and adjustment, flasking, inner and outer coloration of the prosthesis, and other procedures. These complicated processes demand prolonged fabrication periods and numerous visits to the clinic, hence restricting the development and popularization of prosthesis fabrication techniques. In the 1980s, the emergence of CAD/CAM brought a revolutionary transformation to the manufacturing industry and tremendously increased productivity. Since the 1990s, this technique has been gradually introduced to the field of maxillofacial defect rehabilitation, enabling simulative design and rapid manufacturing of prostheses, and thus has remarkably enhanced production efficiency and treatment effects [12–14]. The CAD/CAM-assisted prosthesis manufacturing procedure does not involve uncomfortable facial impression taking, and it has reduced the complexity of wax pattern sculpting. Therefore, production efficiency has been improved with guaranteed treatment effects, and hopefully, this technique will become a regular method of maxillofacial prosthesis production [8, 10].

Digital impressions of maxillofacial soft tissue are fundamental to the intelligentized simulative design of a prosthesis. With the aid of the current digital impression-taking method, data were collected easily and safely, without causing traumatic effects or discomfort to the patient. The mirrored data of the normal side or appropriate data from the database could be used to produce the prosthesis pattern; as a result, the difficulty of the sculpting work could be greatly reduced [4, 5]. Moreover, the precision of the 3D model of the patient's defect area and the adjacent normal region is another issue that is crucial to the whole treatment procedure and is closely related to the final outcome. The 3D model reconstructed from the digital impression data has accurate morphology and is in full accord with the actual morphology of the defect area and the adjacent normal tissue. As a result, the prostheses could be in close contact with the skin surface of the patients [9, 10]. In addition, color information for the soft tissue could also be acquired, making the simulation effects of the prosthesis treatment more realistic in the design procedure, thereby improving the accuracy of the prosthesis design [8].

Researchers have been working on the simulative design and manufacture of maxillofacial prostheses since the 1990s [4–10, 12–17]. In the early methods, it was only used to mirror the data from the normal side to the defect side [4, 6, 12–15]. The surface configuration of the defect area was not considered fully in the design; therefore, large modifications of the wax pattern during the try-in procedure were always needed to adjust the contact area and the detailed surface characteristics. Afterwards, the definitive prosthesis was completed in the traditional way. With further development, the surface data from the defect area was integrated into the design of wax pattern as a step forward for this technique [8, 9, 16, 17]. These methods can solve the problem of compromised rehabilitation outcomes for handmade prostheses due to the weak sculpting ability of the prosthodontists. However, recontouring of the margins and flasking of the mold also needed to be performed manually after the wax pattern try-in step. Although taking the facial impression has been eliminated from the traditional method, and the complexity of wax pattern sculpturing has been reduced, the advantages of CAD/CAM have not been sufficiently exploited. Until the invention of direct fabrication of silicone prostheses by computer numerically controlled machine or rapid prototyping machines, fabrication of prostheses through resin negative molds fabricated by CAD/CAM is undoubtedly the easiest way to produce prostheses. Thin margins of the prosthesis were represented as cavities in the negative mold, so that frangibility of the pattern edge could be avoided completely. With CAD/CAM negative molds, conventional flasking and investing procedures could be totally eliminated, and the border seal between the prosthesis and the patient’s skin could be improved. As a result, use of this system can eliminate the try-in procedure, reduce the number of patient visits, minimize manual labor, shorten the fabrication time, and improve the final prosthetic treatment effect.

Meanwhile, higher requirements are raised with regard to the detailed design of surface features. Realistically detailed surface features are an important factor in the evaluation criteria of a prosthesis, as well as morphology that is in good accord with the patient’s facial characteristics. It is recommended that the design of detailed surface features of a prosthesis be carefully considered, such as the contour of the palpebral, the facial wrinkles (especially in the canthus area), the texture of the skin, and so forth. This is an aspect of this system that needs to be improved in the future.

After the main question of simulative design and manufacture of the contour and shape of the prosthesis has been resolved, individualized color simulation is another indispensable feature of high-simulative maxillofacial prostheses. Our team has also set out to explore the computer-aided printing technique for the extrinsic coloring of maxillofacial prostheses, aiming to further improve the fidelity, reduce the fabrication time, and simplify the manufacturing procedure.

Limitations of the study should be considered when evaluating this new system. Until now, our work was focused on how to establish the whole system, make its operation more simple and convenient, rely less on personal experience, and spend less time obtaining a more realistic prosthesis. All of the patients were satisfied with the appearance of their prostheses. However, there is still a need for objective investigation of whether the changes brought about by the new technique could make patients more satisfied and prostheses more functional. It is extremely important for future researchers to perform objective, controlled studies on patient satisfaction and functional improvement. We will design a more objective and reliable satisfaction questionnaire for future studies.

## Conclusion

CAD/CAM has revolutionized the field of prosthetics. The rapid simulative rehabilitation system for maxillofacial defects related to war and traumatic injuries is approaching completion. With this system, the fidelity and precision of the prosthesis can be increased, manual labor can be simplified, less fabrication time would be needed, and the definitive rehabilitative effect for maxillofacial defects could be improved. This system could provide an advanced technological solution for the Army in cases of maxillofacial wound and defect rehabilitation.
